# Protection against tuberculosis by a single intranasal administration of DNA-hsp65 vaccine complexed with cationic liposomes

**DOI:** 10.1186/1471-2172-9-38

**Published:** 2008-07-22

**Authors:** Rogério S Rosada, Lucimara Gaziola de la Torre, Fabiani G Frantz, Ana PF Trombone, Carlos R Zárate-Bladés, Denise M Fonseca, Patrícia RM Souza, Izaíra T Brandão, Ana P Masson, Édson G Soares, Simone G Ramos, Lúcia H Faccioli, Célio L Silva, Maria HA Santana, Arlete AM Coelho-Castelo

**Affiliations:** 1 Núcleo de Pesquisas em Tuberculose, Faculdade de Medicina de Ribeirão Preto, Universidade de São Paulo, São Paulo, Brazil; 2 Departamento de Processos Biotecnológicos, Faculdade de Engenharia Química, Universidade Estadual de Campinas, São Paulo, Brazil; 3 Departamento de Análises Clínicas, Toxicológicas e Bromatológicas, Faculdade de Ciências Farmacêuticas de Ribeirão Preto, Universidade de São Paulo, São Paulo, Brazil; 4 Departamento de Patologia, Faculdade de Medicina de Ribeirão Preto, Universidade de São Paulo, São Paulo, Brazil

## Abstract

**Background:**

The greatest challenges in vaccine development include optimization of DNA vaccines for use in humans, creation of effective single-dose vaccines, development of delivery systems that do not involve live viruses, and the identification of effective new adjuvants. Herein, we describe a novel, simple technique for efficiently vaccinating mice against tuberculosis (TB). Our technique consists of a single-dose, genetic vaccine formulation of DNA-hsp65 complexed with cationic liposomes and administered intranasally.

**Results:**

We developed a novel and non-toxic formulation of cationic liposomes, in which the DNA-hsp65 vaccine was entrapped (ENTR-hsp65) or complexed (COMP-hsp65), and used to immunize mice by intramuscular or intranasal routes. Although both liposome formulations induced a typical Th1 pattern of immune response, the intramuscular route of delivery did not reduce the number of bacilli. However, a single intranasal immunization with COMP-hsp65, carrying as few as 25 μg of plasmid DNA, leads to a remarkable reduction of the amount of bacilli in lungs. These effects were accompanied by increasing levels of IFN-γ and lung parenchyma preservation, results similar to those found in mice vaccinated intramuscularly four times with naked DNA-hsp65 (total of 400 μg).

**Conclusion:**

Our objective was to overcome the significant obstacles currently facing DNA vaccine development. Our results in the mouse TB model showed that a single intranasal dose of COMP-hsp65 elicited a cellular immune response that was as strong as that induced by four intramuscular doses of naked-DNA. This formulation allowed a 16-fold reduction in the amount of DNA administered. Moreover, we demonstrated that this vaccine is safe, biocompatible, stable, and easily manufactured at a low cost. We believe that this strategy can be applied to human vaccines to TB in a single dose or in prime-boost protocols, leading to a tremendous impact on the control of this infectious disease.

## Background

*Mycobacterium bovis*, bacilli Calmette-Guérin (BCG) has been widely administered to newborns throughout the world showing to be effective in the prevention of childhood tuberculosis (TB) but not in the reactivation of pulmonary disease or human immunodeficiency virus-associated TB. Development of a more effective, standardized, affordable vaccine with durable activity and fewer side effects has been considered a major international public health priority. The need to develop new vaccines is supported by some recent information: 1- data on the natural history and immunology of infection with *M. tuberculosis*; 2- reanalysis of the role of nontuberculous mycobacteria in protection against TB; 3- the understanding of BCG limitations; and 4- the development of molecular techniques that have permitted the identification of immunodominant antigens and new methods of antigen delivery. Vaccine types under investigation against TB include attenuated or enhanced live whole-cell, inactivated whole-cell, subunit, virus-vectored and DNA vaccines, followed by several immunization strategies, as prime-boost protocols. Several of these candidate vaccines have demonstrated activity in animal models that is equal or superior to that of BCG, and trials in human subjects are currently under way [1].We have previously demonstrated that a DNA vaccine encoding the mycobacterial 65-kDa heat shock protein (DNA-hsp65) protected mice and guinea pigs from challenge with a virulent strain of *M. tuberculosis *[2] and cured previously infected mice when administered as naked DNA by intramuscular injection [3]. We have also shown that the therapeutic use of DNA-hsp65 in combination with antimycobacterial drugs shortens the duration of the TB treatment, improves the treatment of latent TB infection, and is effective against multi-drug resistant TB [4].

In spite of some isolated negative experimental results [5], DNA vaccines have in general proved to be safe and well tolerated in preclinical and clinical studies [6]. The naked plasmid DNA molecules have not caused any adverse effects on the biochemical and hematological blood values and have caused neither detectable organ pathology nor systemic toxicity [7]. In addition, there has been no evidence of autoimmunity, development of anti-nuclear or double-stranded DNA antibodies, or plasmid DNA (pDNA) integration into chromosomes [7-10]. Although this approach has been shown to be highly effective in several experimental models, in general a large amount of pDNA administered several times is required to induce this protective immune response. The injection of naked pDNA could lead to its degradation resulting in a reduced transfection efficiency into APCs [11]. Moreover, in early studies of DNA vaccines, experimental data showed that rodents injected with foreign genes expressed antigens, produced antibodies, showed cell-mediated immune responses and achieved protective and long-lasting immunity [12]. However, as these vaccines moved into primate studies and human safety trials, excitement waned. In primate trials, naked DNA failed to generate a successful response [13]. Similar results were observed in our own studies: When we tested the ability of the naked DNA-hsp65 preparation to prevent TB infection in cattle, we did not observe the immunogenicity seen in our mouse model (unpublished data). We have therefore pursued various strategies that allow the use of lower amounts of pDNA with the preservation of the immune protective effects and a simplified vaccination scheme.

Recent approaches to improving the performance of DNA vaccines involves the use of a wide range of adjuvants and delivery systems [14]. Our group has been working on an improvement of DNA-hsp65 vaccination using a drug delivery system. We have previously shown that the intramuscular injection of PLGA [Poly(lactic-co-glycolic acid)] microspheres containing 30 μg of DNA has controlled TB in mice and guinea-pigs [2,15]. Another strategy for DNA delivery is the utilization of cationic liposomes. Since the description of liposome in 1965 by Bangham *et al*. [16], several studies have demonstrated the feasibility of this structure as a delivery system for drugs, peptides, proteins, and DNA. Currently, liposomes are recognized as efficient immunoadjuvants, improving the immune response to various antigens [17-19]. As drug carriers, liposomes are biocompatibile, easy to prepare, and have several formulations that have been approved for clinical application [20]. Gregoriadis and colleagues [21] developed a functional cationic liposome entrapping a DNA vaccine encoding the S (small) region of the hepatitis B surface antigen (HBsAg), that induced an effective immune response. These liposomes had a 16:8 μmol EPC:DOPE (egg phosphatidylcholine:1,2-dioleoyl-*sn*-glycero-3-phosphoethanolamine) ratio and the cationic lipid DOTAP (1,2-dioleoyl-3-trimethylammonium-propane) in the range of 4 to 8 μmol, with the mean diameter of 650 nm. Nonetheless, when the DNA was complexed on the surface of liposomes with the above-mentioned composition, the resulting liposomes were too large (10–20 μm mean diameter) and could not be used in the *in vivo *assays [22,23]. By making changes to these protocols, we achieved a 10:1 molar charge ratio (cationic lipid:DNA) in our liposomes, which resulted in a mean diameter around 1 μm. These liposomes were evaluated using different immunization routes and doses to detect the level of protection against *M. tuberculosis *challenge. Our results showed that the cationic liposomes when complexed with DNA-hsp65 (COMP-hsp65) had similar effects to naked DNA regarding protection against TB. Two main advantages of our new formulation are the 16-fold reduction of the amount of DNA used and the one-time administration by a non-invasive intranasal route. Remarkably, histological examination of the lungs in animals immunized with COMP-hsp65 has revealed minimal granulomatous lesions with evidence of lesion healing and airway remodeling after challenge. We believe that this strategy can be applied to vaccination against TB in a single dose or in prime-boost protocols, with the potential to positively impact the control of this infectious disease.

## Results

### Liposomes are cationic, with different morphologies and appropriate mean size for use in *in vivo *assays

Table 1 presents the physico-chemical properties of cationic liposomes containing entrapped DNA (ENTR-hsp65) or with DNA complexed on their surface (COMP-hsp65). The number-weighted mean diameter and size distribution were obtained by photon correlation spectroscopy (PCS) and dynamic light scattering as previously described [24,25]. An evaluation using number-weighted mean diameter and distribution demonstrated that empty (water-containing) liposomes presented a main population of 247.47 ± 86.26 nm (93.96%) and a second one with 862.92 ± 140.78 nm (6.04%). The ENTR-hsp65 generated two populations of 244.53 ± 64.05 nm (93.21%) and 985.92 ± 229.12 nm (6.79%). The mean diameter and size distribution similarities between empty and ENTR-hsp65 indicate that the amount of DNA does not modify the size of the particles. A different pattern was observed with COMP-hsp65. In this case, the mean size distribution presented two populations with mean diameters 616.73 ± 152.35 nm (93.4%) and 2749.56 ± 774.90 nm (7.53%). The higher size of COMP-hsp65 suggests the presence of DNA on the surface of liposomes.

**Table 1:** Physico-chemical properties of liposomes

**Liposome type**	**Mean Diameter**	**Zeta Potential**
	**nm ± SD^***(i) ***^(%)^***(ii)***^**	**mV ± SD^***(iii)***^**
**Entrapping DNA-hsp65**	244.53 ± 64.05 (93.21) 985.92 ± 229.12 (6.79)	32.8 ± 4.0
**Complexing DNA-hsp65**	616.73 ± 152.35 (93.4) 2749.56 ± 774.90 (7.53)	27.3 ± 2.3
**Empty**	247.47 ± 86.26 (93.96) 862.92 ± 140.78 (6.04)	26.9 ± 2.4

(i) Mean ± standard deviation (SD). Liposome composition- EPC:DOTAP:DOPE 50:25:25 molar. Liposomes containing DNA-hsp65 at molar charge ratio 1:10 (cationic lipid:DNA). Diameter measurements were done in independent samples: n = 8 for liposomes complexed with DNA-hsp65 and n = 9 for liposomes entrapping DNA-hsp65 and empty liposomes.

(ii) Percentages refer to distribution number-weighted of particles.

(iii) Mean ± standard deviation (SD). Zeta potential measurements in three independent samples (n = 3)

The zeta potential of the structures was similar and showed positive values, indicating that the colloidal structures were cationic (Table 1). These results indicate that for a 10:1 molar charge ratio (cationic lipid:DNA), the association of DNA does not influence the zeta potential. Both the colloidal structures were stable in terms of mean diameter and size distribution during storage at 8°C, for 2 months (data not shown).

**Figure 1:**
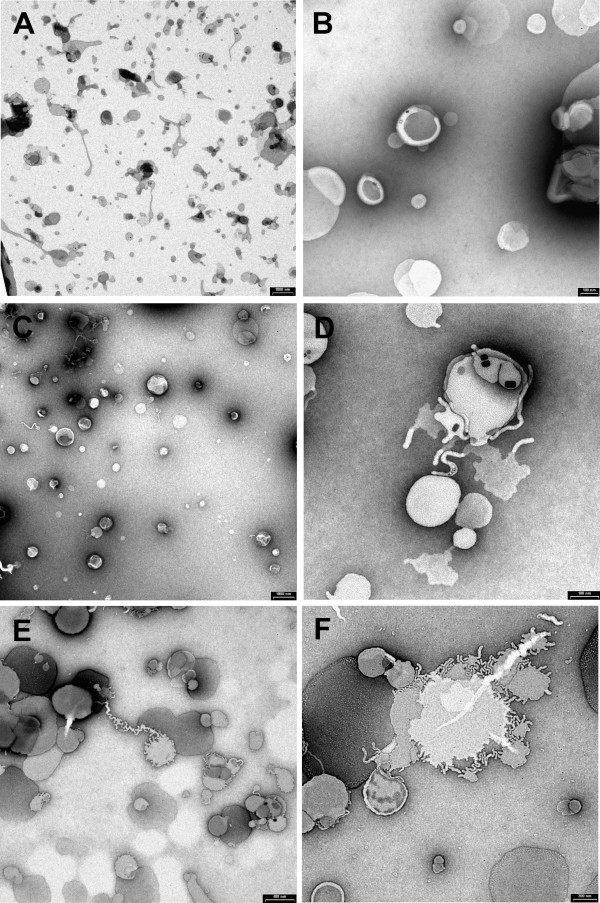
**Negative-staining electron micrographs of liposomes (EPC/DOPE/DOTAP 50:25:25% molar)**. A and B represent empty (water- containing) liposomes; C and D represent liposome entrapping DNA at a 10:1 molar charge ratio (cationic lipid:DNA); E and F represent liposomes complexing DNA at the same molar charge ratio. Bars indicate: A, C, E – 1000 nm; B, D, F – 200 nm.

Transmission electron microscopy using negative-staining techniques were used to investigate the morphology of the structures. Figure 1 represents the electronic micrograph of the three liposome preparations. The results confirm our previous evaluation of mean diameter and size distribution. The empty liposomes (Figures 1A and 1B) appear to be spherical with diameter similar to the main population 250 nm obtained by PCS. The larger structures observed in the Figure 1A could be generated by aggregation due to the dehydration-rehydration process and are in accordance with the second population identified by PCS and dynamic light scattering measurements. In comparison to ENTR-hsp65 (Figures 1E and 1F), the micrographs of COMP-hsp65 (Figures 1C and 1D) show the much higher density of DNA associated with the surface of liposomes. The images also demonstrated that COMP-hsp65 liposomes are larger structures than ENTR-hsp65 liposomes, confirming the mean diameter and size distribution measurements.

### Liposomes are non-cytotoxic when added to cell cultures

**Figure 2:**
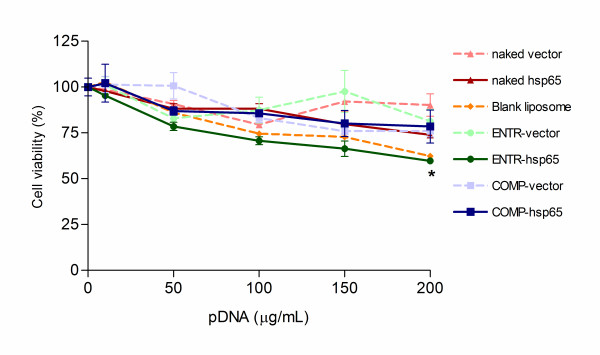
***In vitro *evaluation of cationic liposomes cytotoxicity on J774 macrophage cell line**. Confluent cell populations were incubated with: naked hsp65 or vector; liposome entrapped (ENTR) hsp65 or vector; liposome complexed (COMP) hsp65 or vector. Macrophages were cultured in RPMI medium as the control. The vaccine agents or controls were added to the culture in concentrations ranging from 10–200 μg/mL of DNA, the equivalent of 20–400 μL/mL of liposome, for 24 hours. After treatment, MTT reagent was added to the culture medium and after 4 h of incubation, medium was removed and 100 μL of isopropanol containing HCl 0.1 mol/L was added to the wells to dissolve formazan crystals. Values are the mean ± SD of percent of viable cells compared to the control. The data represent one of three separate experiments performed in quadruplicate. *p < 0.01 were considered significant when compared to naked hsp65 group.

As our main goal was to perform *in vivo *assays with liposome formulations in mice, we evaluated the *in vitro *cytotoxicity of these structures compared to naked DNA (Figure 2). Using the MTT assay, J774 cells were transfected with each of the liposome formulations (ENTR-hsp65 or COMP-hsp65) or with naked hsp65 showing more than 60% of cell viability even with high concentrations of DNA (200 μg/mL, corresponding to 400 μL of liposome). Since our formulations exhibited no difference in toxicity when compared with naked DNA, except for the highest concentration analyzed for Blank (empty) liposome and ENTR-hsp65, these carriers were selected to perform *in vivo *assays with the dose of 50 μg of pDNA.

### Immunization with liposomes carrying DNA-hsp65 induces antibody production of Th1 pattern

**Figure 3:**
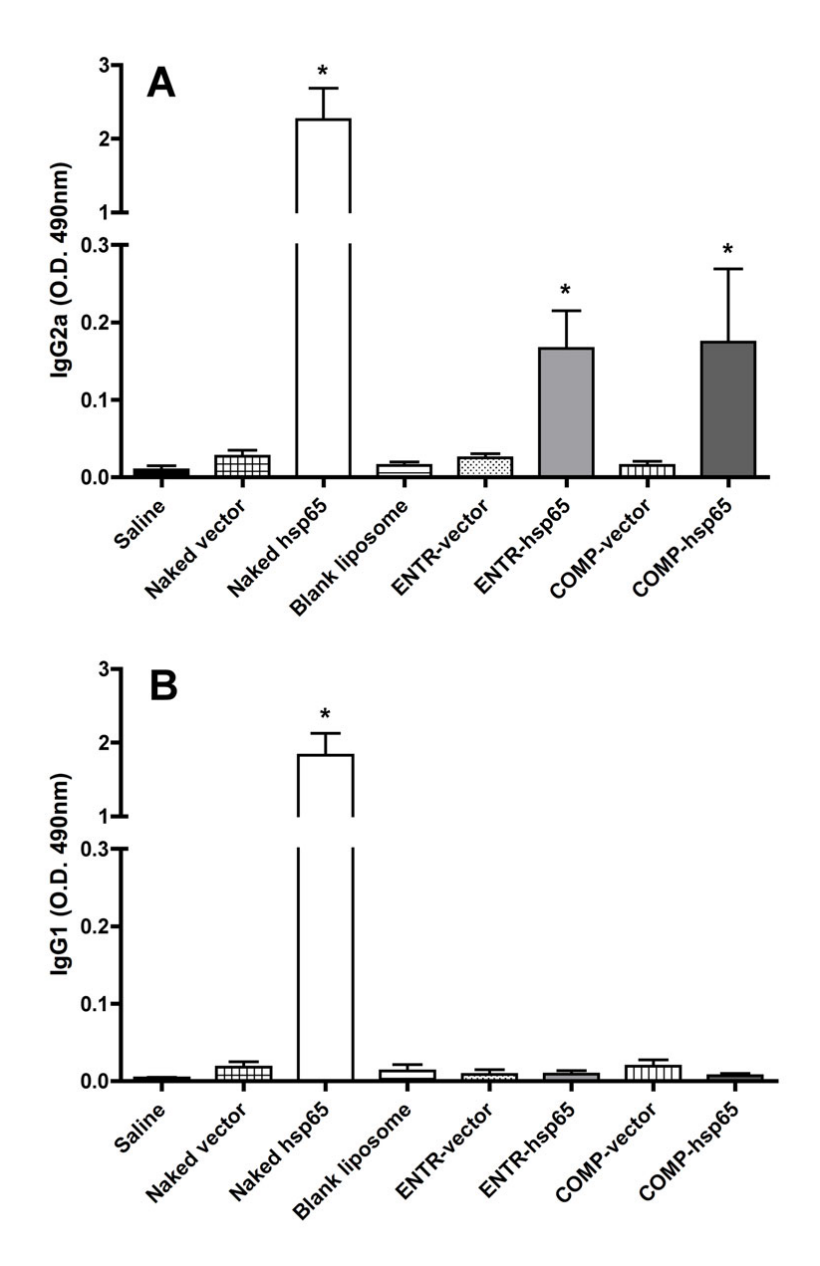
**Anti-hsp65 subtype antibody production in mice immunized with liposomes or naked DNA**. BALB/c mice were immunized by intramuscular injection with 4 doses of 100 μg of naked DNA or a single dose of 100 μL of liposome (corresponding to 50 μg of DNA). Anti-hsp65 antibody levels were evaluated in mice serum by ELISA 30 days after the last immunization. *A*) IgG2a and *B*) IgG1 isotypes. Results are presented by mean ± SD of optical density. *p < 0.05 were considered significant when compared to saline group or their respective controls.

Since the liposomes were not toxic to cells, we analyzed the immunogenic potential of our structures beginning with the evaluation of antibody production after immunization. Thirty days after injection with four doses of naked DNA or a single dose of liposomes, we analyzed the presence of two subtypes of anti-hsp65 antibodies in serum of BALB/c mice. As shown in Figure 3, naked DNA induced a mixed pattern of immune response as previously observed, with IgG1 and IgG2a production. Interestingly, we observed a polarized pattern of antibody production after immunization with cationic liposomes carrying hsp65, with exclusive IgG2a subtype production using only 50 μg of DNA, suggesting a Th1 immune response pattern. However, this IgG2a level was lower than the level induced by naked DNA immunization.

### The route and dose of immunization drive the level of protection against *M. tuberculosis*

**Figure 4:**
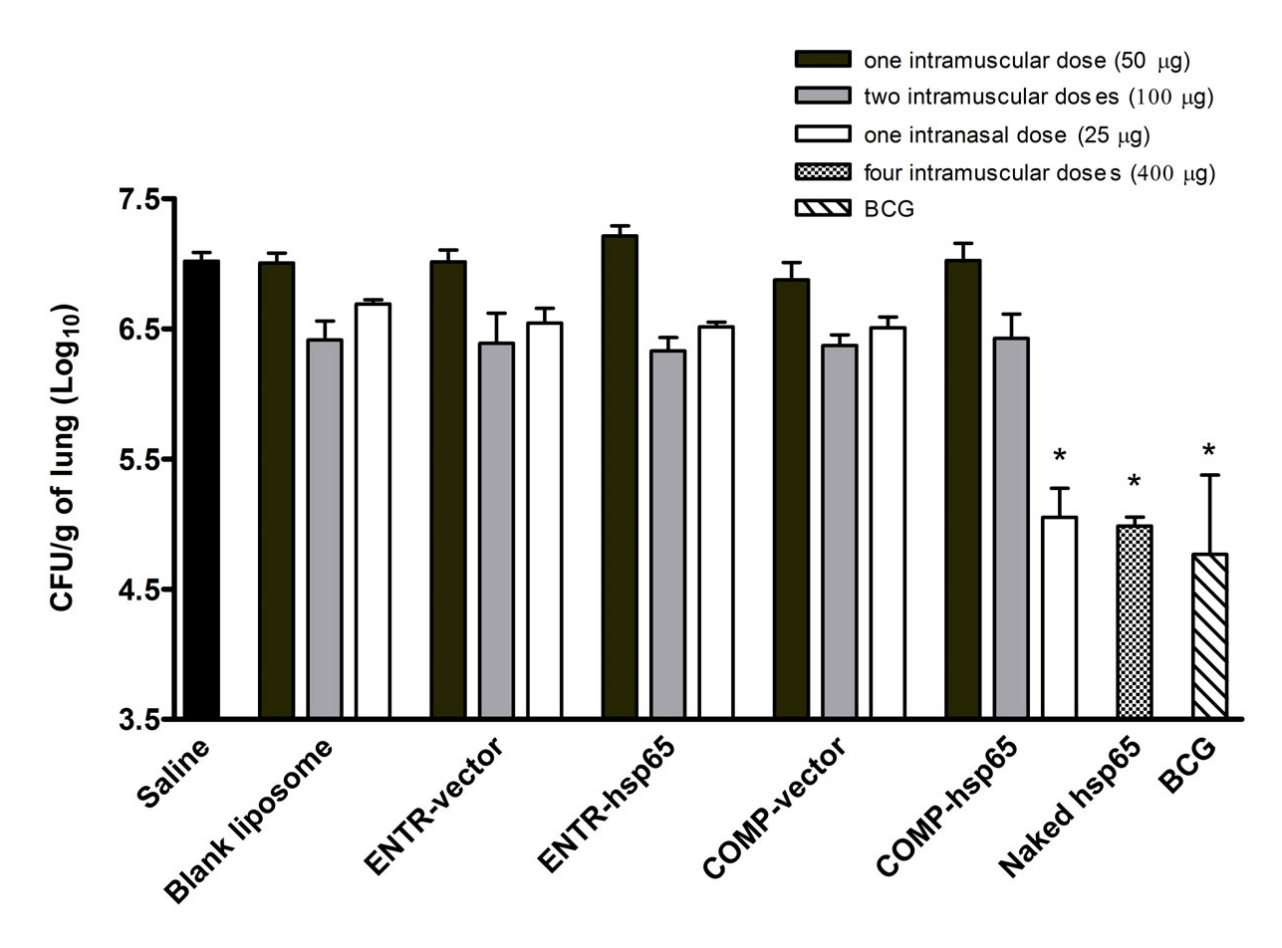
**Determination of *M. tuberculosis *growth in lungs from mice immunized with liposomes by different routes**. BALB/c mice were immunized by: a) a single dose or two doses of liposomes (50 μg of DNA each) by intramuscular injection at a 15-day interval, b) a single dose of liposomes (25 μg) by intranasal instillation, c) four doses of naked DNA (100 μg each) by intramuscular route at a 15-day interval or d) one dose of BCG (Moreau strain) given by subcutaneous injection of about 10^5 ^live bacteria in 100 μl saline. Thirty days after the last dose, mice were challenged intra-tracheally with *M. tuberculosis *H37RV and 30 days post infection we performed CFU analysis. Data represent the mean log_10 _CFU counts ± SD of six mice per group of one of three independent experiments. *p < 0.01 were considered significant when compared to the saline group.

Although a single intramuscular dose of ENTR-hsp65 or COMP-hsp65 induces modulation of the immune system, these formulations do not prevent TB infection in mice when compared with the classic DNA vaccine (Figure 4). The next step was to evaluate the protection against *M. tuberculosis *using different doses and routes of immunization. We attempted to improve the activity of liposomes by using two intramuscular doses of these preparations. This strategy showed a slight colony forming units (CFU) reduction, but overall it was not effective. However, when we delivered COMP-hsp65 intranasally in a single dose (25 μg of DNA) a higher protection was observed, with a significant reduction of 1.97 ± 0.23 log in the bacterial load between the saline group and COMP-hsp65. This CFU reduction was the same as we observed when we vaccinated intramuscularly with four doses (400 μg of total DNA) of naked hsp65 and also similar to results with BCG immunization (Figure 4). Thus intranasal delivery of COMP-hsp65 afforded effective protection in mice using a lower DNA dose. Additionally, four doses of naked hsp65 delivered intranasally did not induce reduction of bacilli number in the lungs (data not shown).

### Protection conferred by COMP-hsp65 was followed by histological examination

**Figure 5:**
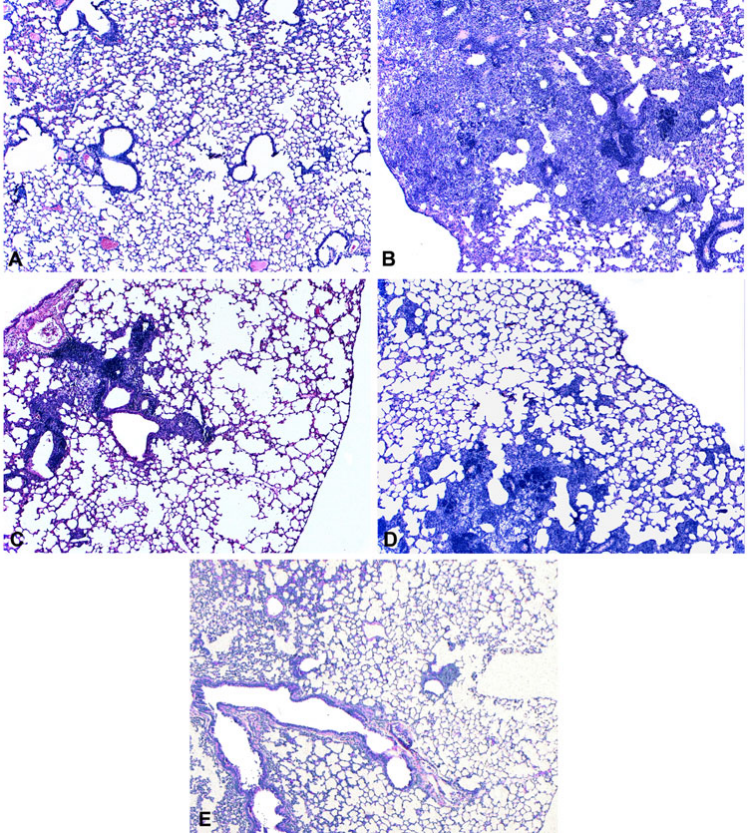
**Comparison of lung parenchyma from mice immunized with COMP-hsp65 or naked hsp65**. Lung sections of mice immunized with one intranasal dose of COMP-hsp65 (25 μg) were compared with lung sections from mice that received four intramuscular doses of naked DNA (400 μg), BCG or saline. Thirty days after the last dose, mice were challenged intra-tracheally with *M. tuberculosis *H37RV and 30 days post infection we performed histological analysis of the lung. (A) non-infected; (B) saline; (C) naked hsp65; (D) COMP-hsp65 and (E) BCG groups. Representative sections of HE staining. Magnifications: ×65.

**Figure 6:**
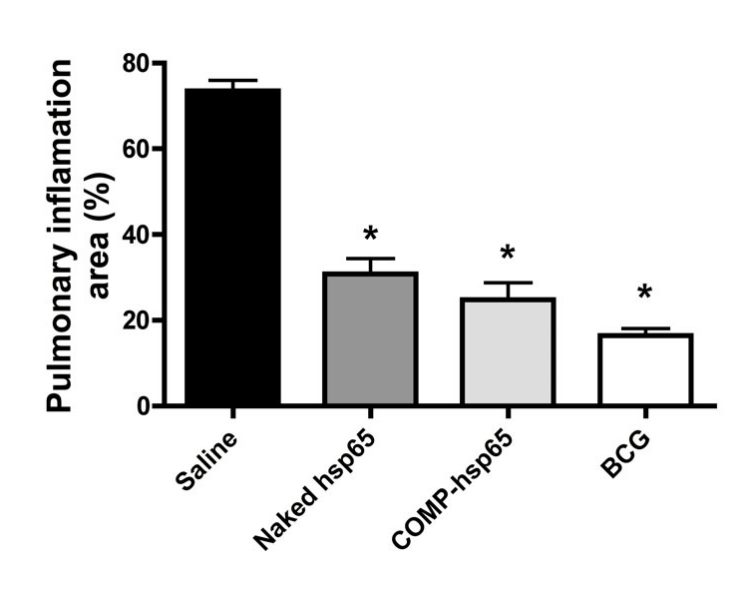
**Morphometric analysis of lung parenchyma from mice immunized with COMP-hsp65 or naked hsp65**. We performed morphometric analysis of lung histology sections. Data represent the mean percent of pulmonary area committed to inflammation ± SD of six mice per group of one of three independent experiments. *p < 0.01 were considered significant when compared to the saline group.

Histological analysis of lung sections from the saline control group (Figure 5) showed tissue damage caused by severe inflammation, with few lymphocyte infiltrations and high numbers of foamy macrophages. In contrast, mice that were immunized with COMP-hsp65 exhibited smaller pneumonic areas with a cell infiltrate containing a predominance of lymphocytes and macrophages. Moreover, the morphometric analysis (Figure 6) showed that 70% of the lung was damaged in the saline control group, while in mice immunized with COMP-hsp65 lung damage was reduced to 30%. These effects of liposome immunization were comparable to those induced by naked hsp65 and BCG.

### The protection of COMP-hsp65 could be related to a Th1 skewed immune response

**Figure 7:**
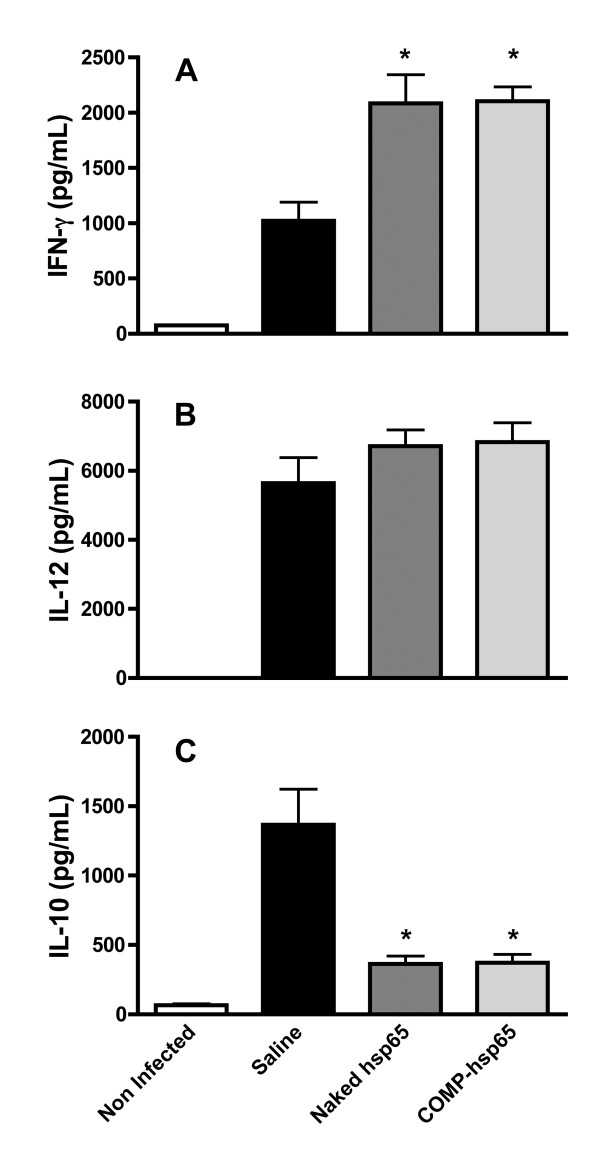
**Cytokine production from the lung tissue of mice immunized with naked DNA-hsp65 or carried by liposome**. Mice were immunized by intramuscular injection with naked hsp65 (400 μg) or by intranasal instillation with COMP-hsp65 (one dose of 25 μg). Thirty days after the last dose, mice were challenged intra-tracheally with *M. tuberculosis *H37RV and 30 days post infection, IFN-γ (**A**), IL-12 (**B**) and IL-10 (**C**) levels were determined by ELISA in lung homogenate. Data represent the mean ± SD of six mice per group of one of three independent experiments. *p < 0.01 were considered significant when compared to the saline group.

Since certain cytokines play an important role in the immune response to *M. tuberculosis*, we investigated the pattern of cytokine production in the lungs of mice from saline controls and mice immunized according to protocols that had shown protection, e.g., naked DNA and COMP-hsp65. When mice were challenged with *M. tuberculosis*, we observed a Th1 cytokine pattern with an increase of IFN-γ, IL-12 and IL-10 production when compared to uninfected animals (Figure 7). However, immunization with naked hsp65 and COMP-hsp65 induced a significant increase of IFN-γ and a significant decrease in IL-10 compared to saline control mice. IL-12 expression was not altered by these immunization protocols. Additionally, we did not observe differences in IL-4 production among the groups (data not shown).

## Discussion

Our group has focused on the heat shock protein produced by *Mycobacterium leprae *(hsp65) as a vaccine antigen against several pathologies including TB, leishmaniasis [26], diabetes, arthritis [3,4,27-29] and cancer. The success of hsp65 vaccination in these different diseases reflects its effectiveness as an immunodominant antigen, and also suggests that it possesses hsp-dependent properties, such as causing the formation of peptide-hsp complexes [30]. When DNA-hsp65 was used to vaccinate mice intramuscularly it elicited an effective immune response only at high DNA doses, which we postulate were necessary because of the loss of a considerable amount of injected pDNA via deoxyribonuclease degradation. To circumvent this problem, drug carriers such as microspheres or liposomes have been developed which protect DNA from extracellular enzymes and could improve DNA vaccination results.

Here we showed that cationic liposomes could be used as delivery system for low doses of DNA-hsp65, causing protection equivalent to that of naked DNA against *M. tuberculosis*. Most important were the findings that the use of liposomes resulted in a 16-fold reduction in the amount of DNA used in the immunization and that this could be administered in a single dose through a non-invasive route.

The physico-chemical characterization of liposomes containing DNA entrapped or complexed on the surface demonstrated that these structures have positive zeta potential. This cationic characteristic supports DNA delivery to the cells since the driving force for the binding of lipid structures to the negatively charged cell membrane is electrostatic [31]. Furthermore, different DNA-liposome association methods produced liposomes with different characteristics of mean diameter and morphology. Our findings are in accordance with the studies of Perrie and colleagues [23], which described the DNA localization in dehydrated-hydrated vesicles obtained by entrapping or complexing methods. The entrapping process distributes the DNA along the bilayers and the complexing concentrates the DNA on the external surface of the vesicle. However the mean size obtained in our study with complexed DNA on the liposome surface was smaller than previously reported [23] due to our modified protocol, rigid temperature control (4°C) and intense vortexing.

*In vitro *studies showed that our liposome preparations are safe with low cytotoxicity even at high concentrations. Also, *in vivo *studies showed that the immunization with liposomes skewed the antibodies production to IgG2a without IgG1 production, suggesting a polarization to Th1 pattern of immune response. This profile is similar to previous data of our group injecting PLGA microspheres carrying DNA-hsp65 plus trehalose 6,6'-dimycolate (an adjuvant derived from cell walls of mycobacteria) by intramuscular route [15]. Results from immunization with naked hsp65 showed a mixed antibody response and levels of IgG2a antibodies than seen with the liposome delivery system. These differences could be related to the amount of DNA, the different doses or to the immunization route. However, the liposomes are the delivery vehicle of choice as they elicited Th1 polarization without the use of additional adjuvant.

Our analysis of different immunization protocols showed that one intramuscular dose (which delivers 50 μg of DNA) did not promote a significant reduction in CFU number (Figure 4). However, all groups immunized twice with liposome exhibited a slight reduction in bacterial growth, suggesting that the constituents of liposomes may have intrinsic immunostimulatory properties. To analyze a new route of delivery, we used the immunization through the nasal mucosa that provides a simple, non-invasive route to deliver DNA vaccines stimulating mucosal immunity [32]. While this worked effectively for our liposome bearing DNA-hsp65, the intranasal delivery of naked DNA was ineffective. Possibly naked DNA was destroyed by physical and chemical mucosal barriers that abrogate the gene transfection including endonucleases, enzymes present on mucus, low pH and fast elimination by ciliate epithelium [33,34], a scenario that can be circumvented by liposomal carriers. Furthermore, cationic liposomes have been proved to enhance immune response against pathogens [32,35] including *M. tuberculosis *[36] when administered intranasally. COMP-hsp65 intranasal instillation conferred a significant impairment in bacilli growth that was comparable to 400 μg of naked DNA injected by intramuscular route.

Since the lipids and molar ratio of both liposomes were similar, the reason that only COMP-hsp65 immunization achieved effective protection could be due to where the DNA resides. The higher exposure of DNA on the liposome surface in COMP-hsp65 may favor the interaction of CpG motifs present in plasmid DNA with receptors such as endogenous toll-like receptor 9 (TLR9) leading to an activation of antigen-presenting cells [37]. Another feature to be considered is that the diameter of the liposomes could influence the macrophage phagocytosis process, as showed by Volle *et al*. [38], which established a relation between the size of synthetic particles and phagocytosis efficacy, a result confirmed in rat alveolar macrophages [39]. Related, we observed that mice immunized with larger particles (COMP-hsp65) caused a significant reduction of CFU from infected lung tissue, suggesting that COMP-hsp65 delivery improved the DNA capture by immune competent cells. However, additional studies are required to test this hypothesis.

In addition to protecting against bacterial growth, new vaccines need to be evaluated for their ability to maintain the integrity of the tissues involved in the pathology. In our study, COMP-hsp65 vaccination preserved the pulmonary parenchyma (Figure 5) and reduced tissue inflammation (Figure 6). Therefore, liposomes complexed with DNA-hsp65 showed the ability to stimulate the immune system in a manner that resulted in bacilli reduction and lung preservation. These effects could be due to cytokines present in the pulmonary parenchyma, as high levels of IFN-γ were detected in mice immunized with both naked hsp65 and COMP-hsp65 (Figure 7). This finding suggests that COMP-hsp65 is a promising vaccine, since a Th1 immune response could be important for the development of protective immunity against *M. tuberculosis *infection [29,40-42]. On the other hand, production of the regulatory cytokine IL-10 was reduced in vaccines that conferred effective protection against *M. tuberculosis *infection (naked hsp65 and COMP-hsp65), when compared to non-immunized mice. This is particularly interesting because the role of IL-10 in TB is controversial, since it has been reported that IL-10^-/- ^mice are not protected from infection [43,44] while others showed that increases of IL-10 expression leads to a down regulation of the immune response against TB [45,46]. Indeed, our group recently showed that the evaluation of many immunological parameters is necessary to understand the mechanisms underlying the immune response against TB [47,48]. In our model, it seems that a combination of the increase of IFN-γ expression and a decrease of IL-10 expression improves the immune response leading to bacilli reduction and lung parenchyma preservation.

The utilization of cationic liposomes as a drug delivery system represents an advance for vaccine technology, which has been growing in recent years. Liposomes delivering amphotericin B is used in the therapy of visceral leishmaniasis patients with great efficacy [49]. Okada and colleagues showed 100% of survival in a cynomolgus monkey model infected with *M. tuberculosis*, using BCG plus DNA-hsp65 vaccine carried by liposomes [50]. Moreover, several articles pointed out the effects of DNA-hsp65 immunization in different protocols and a study has been done to achieve an effective, cost-convenient and safest vaccination scheme (Souza *et al*., in press). Additionally, two DNA vaccines for veterinary use (horse and salmon) are in the market and there are several reports showing the safety of DNA vaccines when tested in preclinical and clinical trials [51]. We recently tested DNA-hsp65 vaccine in a phase I/II clinical trial in cancer patients and the results showed no toxicity or autoimmunity reactions (Michaluart *et al*., in press). Considering that liposomes are currently used in medical products and DNA vaccines are under development, their combination in TB vaccination offers a promising approach to fighting this disease.

## Conclusion

Here we showed effective protection against TB with a single immunogen delivered by a safe and efficient carrier system without any additional adjuvant. We achieved a 16-fold reduction in the plasmid DNA amount administered in only one dose with the additional advantage of using a non-invasive route of administration (intranasal route). The data presented here show the importance of the proper association of the delivery method and the optimization of the route and dose used for immunization.

## Methods

### Plasmid derivation

The naked hsp65 construct was derived from the pVAX1 vector (Invitrogen, Carlsbad, CA, USA). The 3.3 kb fragment of *M. leprae *hsp65 was subcloned into Bam HI and Not-I restriction sites (Gibco BRL, Gaithersburg, MD, USA). pVAX1 uses a CMV from intron A as a promoter. The parental vector was used as control. Plasmid DNA was obtained from transformed DH5α *E. coli *cultured in LB liquid medium (Gibco BRL, Gaithersburg, MD, USA) containing kanamicin (50 μg/mL). The plasmids were purified using the Endofree Plasmid Giga kit (Qiagen, Valencia, CA, USA). Plasmid concentration was determined by spectrophotometry at the wave lengths 260 and 280 nm using the Gene Quant II apparatus (Pharmacia Biotech, Buckinghamshire, UK).

### Liposomes preparation and characterization

#### Lipids

Egg phosphatidylcholine (EPC), 1,2-dioleoyl-*sn*-glycero-3-phosphoethanolamine (DOPE) and 1,2-dioleoyl-3-trimethylammonium-propane (DOTAP) were purchased from Avanti Polar Lipids.

The cationic liposomes were prepared in three steps: (i) preparation of liposomes according to Bangham's method [16], (ii) dehydration by lyophilization and (iii) hydration to obtain dehydrated-hydrated vesicles (DRVs) described by Kirby and Gregoriadis [52]. Briefly, the required amounts of all lipid stock solutions in chloroform (EPC/DOPE/DOTAP 50/25/25% molar) were mixed and dried to a thin film using a rotary evaporator in a 650 mmHg vacuum for 1 hour. The dried lipid film was hydrated with water at 30°C above its phase transition temperature. The liposomes were extruded through two stacked polycarbonate membranes (100 nm nominal diameter) 15 times at a nitrogen pressure of 12 kgf/cm^2^.

##### Cationic liposomes entrapping plasmid DNA

DNA (pVAX-hsp65) in water solution was mixed with the extruded liposomes, frozen, and freeze-dried overnight. Controlled rehydration of the dry powders with saline solution (NaCl 0.9%) resulted in the formation of liposomes entrapping the DNA referred as ENTR-hsp65 [23].

##### Cationic liposomes complexed with plasmid DNA

The complexed structure containing higher DNA density on the surface (COMP-hsp65) was obtained through a modification of the protocol described by Perrie and Gregoriadis [23]. Preformed empty dehydrated-hydrated liposomes (1.44% NaCl) were complexed with water resuspended DNA reaching a 0.9% NaCl final concentration. The complexing was carried out at 4°C under vortex for size control.

#### The cationic lipid

DNA molar charge ratio of 10:1 was used in both liposomal structures. The biophysical characterization of liposomes was assessed as follows:

##### Average hydrodynamic diameter and distribution

The average hydrodynamic diameter and size distribution were determined by Photon Correlation Spectroscopy (PCS) and dynamic laser light scattering (Malvern Autosizer 4700) using a Ne-He laser and measurements were taken at a scattering angle of 90°. Particle diameter was calculated from the translational diffusion coefficient by using the Stokes-Einstein equation: d(H) = (kT)/(6πηD)

where: d(H) is the hydrodynamic diameter, D is the translational diffusion coefficient, k is the Boltzmann's constant, T is the absolute temperature, and η is the viscosity. The mean diameter and distribution of particle sizes were estimated by CONTIN algorithm analysis. Results were calculated in triplicate and expressed by the intensity of scattered light and converted to number-weighted mean diameter and size distribution automatically by the software of the equipment.

##### Zeta Potential

The zeta potential was measured on a Zetasizer 3000 – Malvern by diluting empty cationic liposomes or those containing DNA in appropriate volume of saline solution (NaCl 0.9%) at pH 6.4, 25°C.

##### Morphology

Transmission electron microscopy and the negative staining method were used to visualize the morphology of the lipid structures. Carbon-coated 200 mesh copper grids with collodion (parloidin with cellulose acetate) film were used. Each liposome preparation was diluted to 1 mM total lipid and then applied to the carbon grid. After incubation for 5 minutes at room temperature, the excess was blotted. One drop of uranyl acetate (1% w/w in saline solution) was added to the carbon grid and incubated 1 minute at room temperature before the excess was blotted and air-dried. A Carl Zeiss CEM 902 microscope, equipped with a Castaing-Henry-Ottensmeyer energy filter was used. The images were obtained using a CCD camera (Proscan).

All formulations were tested for endotoxin levels with QCL-1000 Limulus amebocyte lysate. The levels found were under 0.01 EU/mL.

### *In vitro *cytotoxicity assay

The standard 3-(4,5-diethylthiazoyl-2-yl)-2,5-diphenyltetrazolium bromide (MTT) colorimetric cytotoxicity assay was used [53]. J774-macrophage cells were grown in RPMI medium at 10^6 ^cells/mL and added to 96-well cell culture plates at 10^5 ^cells/well. The cells were incubated for 24 h at 37°C, with 5% CO_2_. Serial dilutions of the liposomes or naked DNA in RPMI medium were added as described below. Cells were incubated with vaccine preparations for 24 h and then 100 μL MTT reagent (5 mg/mL in RPMI) was added to each well. MTT was allowed to incubate with the cells for 4 h. The supernatant were aspirated and 100 μL of isopropanol plus HCl 0.1 mol/L was added to each well. After plate agitation the absorbance of the solubilized compounds was read on spectrophotometer set at 570 nm. Cell survival at the end of treatment was calculated as a percentage of the control cells (macrophages incubated only in the medium). All assays were performed in quadruplicate.

### Animals

Female 6-week-old BALB/c mice were obtained from the animal facility of Faculdade de Ciências Farmacêuticas – Universidade de São Paulo. All experiments were approved and conducted in accordance with the guidelines of the Animal Care Committee of the University. Infected animals were kept in biohazard facility of Laboratory Biosafety Level 3 and housed in cages within a laminar flow safety enclosure under standard conditions.

### Immunization procedures

Immunization was performed by one of the following treatments, using six animals per group. For BCG immunization, one dose of Moreau strain was given by subcutaneous injection of 10^5 ^live bacteria in 100 μl of saline. For naked DNA vaccination, the plasmid with DNA-insert (DNA-hsp65) was administered by intramuscular injection of 50 μg in 50 μL of saline solution into each quadriceps muscle in four occasions at two-week intervals (total dose of 400 μg of plasmid DNA). For liposomes vaccination, mice received one or two doses of 50 μg of DNA (equivalent of 100 μL of liposome) by intramuscular injection. For intranasal delivery of liposomes, animals were lightly anesthetized with tribromoethanol 2,5% (Across Organics) and 50 μL of liposome (25 μg of DNA)/mouse was administered dropwise to external nostrils of the mice (25 μL per nostril) with a fine pipette tip. Additionally control animals received parental vector plasmid pVAX1 entrapped (ENTR) or complexed (COMP) in liposomes, or empty liposomes (blank).

### Antibody evaluation

Serum from vaccinated or control mice were collected 30 days after the last dose of each immunization scheme. To assess antigen-specific antibody levels, 96-well plates (Maxisorp Nunc-Immuno plates) were treated with 0.1 mL of purified protein (5 μg/mL) in coating solution (Na_2_CO_3 _14.3 mM, NaHCO_3 _10.3 mM, NaN_3 _0.02%, pH9.6), incubated at 4°C overnight, and then blocked with 1% BSA in PBS for 60 min at 37°C. Serum samples were applied in serial ten-fold dilutions from 1:10. After incubating the plates for 2 h at 37°C, anti-mouse biotin-conjugated IgG1 or IgG2a (A85-1 and R19-15 respectively, from Pharmingen, San Diego, CA, USA) were added for the detection of specific antibodies. After washing, plates were incubated at room temperature for 30 min with StreptAB kit (Dako, Carpinteria, CA, USA). The detection of bound antibodies was conducted by OPD substrate (Sigma, St Louis, USA) and the reaction was stopped by the addition of 50 μL of a 16% solution of sulfuric acid. Optical density was measured at 490 nm.

### Experimental infection with *M. tuberculosis*

The H37Rv strain of *M. tuberculosis *(American Type Culture Collection, Rockville, MD) was grown in 7H9 Middlebrook broth (Difco Laboratories, Detroit, MI) for seven days. The culture was harvested through centrifugation and the cell pellet was resuspended in sterile phosphate buffered saline (PBS) and vigorously agitated. The homogeneous suspension was filtered through 2-μm filters (Millipore, Bedford, MA). Viability of the *M. tuberculosis *suspension was pretested with fluorescein diacetate (Sigma, Saint Louis, MO) and ethidium bromide. Thirty days after the last immunization the mice were challenged with *M. tuberculosis*. An anterior midline incision was made to expose the trachea. A 30-gauge needle attached to a tuberculin syringe was inserted into the trachea and intratracheal dispersion was used to introduce 10^5 ^viable CFU of *M. tuberculosis *H37Rv in 100 μl of PBS into the lungs [27]. Thirty days after the *M. tuberculosis *challenge the mice from all groups were euthanized. Control mice received only intratracheal PBS.

### Determination of *M. tuberculosis *CFU in lungs

The rescue of *M. tuberculosis *was performed as described previously [3]. Briefly, the number of live bacteria was determined by extracting the lower and medium right lobes of the lung, washed with sterile PBS, followed by plating 10-fold serial dilutions of homogenized tissue on Middlebrook 7H11 agar media (Difco) [supplemented with 0.2% (v/v) glycerol and 10% (v/v) bovine fetal serum], counting colonies after 28 days at 37°C. The colony-forming units (CFU) are expressed as log_10 _of CFU/g lung.

### Histology and morphometric analysis of lung parenchyma

At 30 days post-*M. tuberculosis *infection, the upper right lobe of each mouse lung was removed and fixed in 10% formalin. Paraffin blocks were prepared and then sectioned for light microscopy. Sections (5 μm each) were stained with hematoxylin & eosin (HE). Slides were evaluated using a Leitz Model Aristoplan microscope (Germany) connected to a Leica Model DFC280 color camera (Heerbrugg, Germany) linked to a PC computer. To perform morphometric analysis of lung parenchyma, an integrating eyepiece with a coherent system made of a 100-point grid consisting of 50 lines of known length was coupled to the slides and evaluated through light microscopy. Volume fraction of collapsed and normal pulmonary areas was determined by point-counting technique, made at a magnification of ×400 across 10 random non-coincident microscopic fields. Points falling on tissue area were counted and divided by the total number of points in each microscopic field. Thus data were reported as the fractional area of pulmonary tissue [54].

### Cytokine evaluation

For cytokine measurements, the entire left lobe of lung was removed on day 30 post-*M. tuberculosis *infection. Tissue was homogenized in 2 ml of RPMI 1640, centrifuged at 450 × *g *and the supernatant was stored at -70°C until assayed. Commercially available enzyme-linked immunosorbent assays with capture and biotinylated monoclonal antibodies were used to measure IFN-γ (R4-6A2, XMG1.2), IL-4 (11B11, BVD6-24G2), IL-10 (JES5-2A5, SXC-1) and IL-12 (9A5, C17.8) (Pharmingen, San Diego, CA). The cytokine levels were measured according to the manufacturer's instructions with sensitivities >10 pg/ml.

### Statistical analysis

The data were represented as mean ± SD (n = 6) and analyzed using GraphPad Prism version 4.03 for Windows (GraphPad Software, San Diego, CA). The data were compared using Mann-Whitney non-parametric test.

## Authors' contributions

RSR, LGdlT, FGF, APFT, CRZ–B, DMF, PRMS, ITB, APM participated in the design of the study and *in vivo *and *in vitro *experiments. RSR, LGdlT carried out the liposomes formulations. RSR, LGdlT, FGF, APFT, CRZ–B drafted the manuscript and performed the statistical analysis. EGS, SGR, LHF, CLS, MHAS, AAMC–C conceived of the study, participated in its design and coordination and helped to draft the manuscript. All authors read and approved the final manuscript.
